# Update on *Cryptosporidium* spp.: highlights from the Seventh International *Giardia* and *Cryptosporidium* Conference

**DOI:** 10.1051/parasite/2020011

**Published:** 2020-03-13

**Authors:** Giovanni Widmer, David Carmena, Martin Kváč, Rachel M. Chalmers, Jessica C. Kissinger, Lihua Xiao, Adam Sateriale, Boris Striepen, Fabrice Laurent, Sonia Lacroix-Lamandé, Gilles Gargala, Loïc Favennec

**Affiliations:** 1 Department of Infectious Disease & Global Health, Cummings School of Veterinary Medicine at Tufts University North Grafton 01536 MA USA; 2 Spanish National Centre for Microbiology 28220 Majadahonda Spain; 3 Institute of Parasitology, Biology Centre CAS 370 05 České Budějovice Czech Republic; 4 Faculty of Agriculture, University of South Bohemia in České Budějovice 370 05 České Budějovice Czech Republic; 5 Cryptosporidium Reference Unit, Public Health Wales SA2 8QA Swansea UK; 6 Swansea Medical School, Swansea University SA2 8PP Swansea UK; 7 Center for Tropical and Emerging Global Diseases, Institute of Bioinformatics and Department of Genetics, University of Georgia Athens 30602 GA USA; 8 College of Veterinary Medicine, South China Agricultural University Guangzhou 510642 Guangdong PR China; 9 Department of Pathobiology, School of Veterinary Medicine, University of Pennsylvania 380 South University Avenue Philadelphia 19104 PA USA; 10 INRAE, Université François Rabelais de Tours, Centre Val de Loire, ISP, Laboratoire Apicomplexes et Immunité Mucosale 37380 Nouzilly France; 11 French National Cryptosporidiosis Reference Center, Rouen University Hospital 1 Rue de Germont 76031 Rouen Cedex France; 12 EA 7510, UFR Santé, University of Rouen Normandy, Normandy University 22 Bd. Gambetta 76183 Rouen Cedex France

**Keywords:** *Cryptosporidium*, Cryptosporidiosis, Epidemiology, Genomics

## Abstract

While cryptosporidiosis is recognized as being among the most common causes of human parasitic diarrhea in the world, there is currently limited knowledge on *Cryptosporidium* infection mechanisms, incomplete codification of diagnostic methods, and a need for additional therapeutic options. In response, the Seventh International *Giardia* and *Cryptosporidium* Conference (IGCC 2019) was hosted from 23 to 26 June 2019, at the Rouen Normandy University, France. This trusted event brought together an international delegation of researchers to synthesize recent advances and identify key research questions and knowledge gaps. The program of the interdisciplinary conference included all aspects of host-parasite relationships from basic research to applications to human and veterinary medicine, and environmental issues associated with waterborne parasites and their epidemiological consequences. In relation to *Cryptosporidium* and cryptosporidiosis, the primary research areas for which novel findings and the most impressive communications were presented and discussed included: *Cryptosporidium* in environmental waters, seafood, and fresh produce; Animal epidemiology; Human cryptosporidiosis and epidemiology; Genomes and genomic evolution encompassing: Comparative genomics of *Cryptosporidium* spp., Genomic insights into biology, Acquiring and utilizing genome sequences, Genetic manipulation; Host-parasite interaction (immunology, microbiome); and Diagnosis and treatment. High quality presentations discussed at the conference reflected decisive progress and identified new opportunities that will engage investigators and funding agencies to spur future research in a “one health” approach to improve basic knowledge and the clinical and public health management of zoonotic cryptosporidiosis.

## Abstract (fr)

Bien que la cryptosporidiose soit reconnue comme l’une des premières causes de diarrhée parasitaire humaine dans le monde, la connaissance des mécanismes de l’infection par *Cryptosporidium* est limitée, la codification des méthodes diagnostiques est incomplète et des options thérapeutiques supplémentaires sont requises. En réponse à cette situation, la Septième Conférence Internationale sur *Giardia* and *Cryptosporidium* (IGCC 2019) s’est tenue du 23 au 26 juin 2019, à l’Université de Rouen-Normandie, France. Cet événement renommé a rassemblé une délégation internationale de chercheurs pour faire la synthèse des avancées récentes et identifier les principaux thèmes de recherche et les lacunes dans les connaissances. Le programme de cette conférence interdisciplinaire comprenait tous les aspects des relations hôte-parasite, de la recherche fondamentale aux applications à la médecine humaine et vétérinaire, ainsi que les questions environnementales liées aux parasites d’origine hydrique et leurs conséquences épidémiologiques. En ce qui concerne *Cryptosporidium* et la cryptosporidiose, les principaux domaines de recherche pour lesquels de nouvelles découvertes et les communications les plus impressionnantes ont été présentées et discutées comprenaient : *Cryptosporidium* dans les eaux environnementales, les fruits de mer et les produits frais ; Épidémiologie animale ; Cryptosporidiose et épidémiologie humaine ; Génomes et évolution génomique englobant : Génomique comparative des *Cryptosporidium* spp., Perspectives génomiques en biologie, Acquisition et utilisation des séquences du génome, Manipulation génétique ; Interaction hôte-parasite (immunologie, microbiome) ; Diagnostic et traitement. Les présentations de grande qualité discutées à la conférence ont fait état de progrès décisifs et ont permis de cerner de nouvelles possibilités qui inciteront les chercheurs et les organismes de financement à stimuler la recherche future dans une approche « une seule santé » afin d’améliorer les connaissances de base et la gestion clinique et de santé publique de la cryptosporidiose zoonotique.

## Introduction

The Seventh International *Giardia* and *Cryptosporidium* Conference (IGCC) was held in Rouen, France, from 23 to 26 June 2019. The conference, which covered various research areas on *Cryptosporidium* and cryptosporidiosis, including epidemiology, genomics and genome evolution, genetic manipulation, host parasite interaction, and diagnosis and treatment, was attended by 237 researchers from more than 100 research teams from 34 countries. We would like to highlight here the most impressive communications and novel findings presented at the IGCC-7.

## *Cryptosporidium* in environmental waters, seafood, and fresh produce

*Cryptosporidium* spp. are largely recognized as common causes of water- and food-borne outbreaks of diarrheal illness globally [11, 30]. Waterborne transmission is a major route in the epidemiology of the parasite and is thus a serious public health concern. As a reflection of the interest in the transmission of cryptosporidiosis, a significant number of studies presented at IGCC-7 focused on the detection and quantification of *Cryptosporidium* oocysts in surface (Cho et al., p. 56 in [29]; Hatalová et al., p. 58 in [29]; Wells et al., p. 130; p. 164 in [29]; Cirkovic et al., p. 132 in [29]), recreational (Chalmers et al., p. 185 in [29]), and waste water matrices (Zahedi et al., p. 126 in [29]; Cervero-Aragó et al., p. 129 in [29]). Most of these surveys were conducted using different methodological approaches (concentration, purification, and identification), in spite of the existence of standard methods (*e.g.*, ISO 15553, USEPA 1623, and 1693). More effort should be devoted to understanding the factors (*e.g.*, cost, availability, feasibility) behind the motivation not to use procedures approved for routine or regulatory monitoring.

Livestock (mainly cattle) and wildlife (deer and migrating geese) were demonstrated as relevant contributors to zoonotic *Cryptosporidium* oocysts in source water catchments intended for human consumption (Wells et al., p. 130; p. 164 in [29]), but wildlife control measures may be difficult to implement due to legal and conservation issues.

Importantly, new technologies such as ceramic candle filters (Abeledo-Lameiro et al., p. 142 in [29]) and alternative sampling strategies including aquatic biofilms (Jellison et al., p. 122 in [29]; Ortega et al., p. 124 in [29]), adapted methods for use in swimming pool waters (Chalmers et al., p. 185 in [29]), qPCR assays applied to partially purified oocysts on microscope slides (Elwin et al., p. 133, in [29]) and next generation sequencing (NGS) approaches (Zahedi et al., p. 126 in [29]) have been developed to sample and detect the parasite in different water matrices.

Large-scale foodborne outbreaks of cryptosporidiosis have been documented globally in recent years [30]. Therefore, increasing attention is being paid to the detection of *Cryptosporidium* oocysts in fresh produce including lettuce, parsley, coriander, fruits, and sprout seeds (Berrouch et al., p. 135 in [29]; Bartošová et al., p. 136 in [29]). Standard methods for food are limited to leafy greens and berry fruits (ISO 18744; BMA19A). It was evident in these surveys that a variety of methods were used, making it difficult to directly compare results among different research groups. The use of qPCR increased detection sensitivity, but a lack of genotyping and sub-genotyping data makes it impossible to fully evaluate the public health relevance of these findings. Edible filtrating molluscs, including oysters, have been proposed as potential sources of human cryptosporidiosis (Santos et al., p. 57 in [29]), although no recognized outbreaks have been documented so far [21]. Interestingly, small crustaceans of the genus *Artemia* have been successfully used as a bio-indicator for the presence of zoonotic *Cryptosporidium* oocysts in water reservoirs (Hatelová et al., p. 58 in [29]).

## Animal epidemiology

Molecular tools are increasingly being used for genotyping and subtyping of *Cryptosporidium* spp. originating from farm, domestic and wild animals, and for monitoring the sources of infections and transmission routes. Although Sanger sequencing technology still prevails, the use of NGS has increased in recent years. NGS is advantageous for detecting mixed infections, as these technologies are able to identify more subtypes infecting the same host than those revealed by Sanger sequencing (Santín et al., p. 165 in [29]). It is now also possible to amplify DNA from individually purified *Cryptosporidium* oocysts, a method that has been used to ascertain the genetic population of the parasite at the herd/farm level or even within individuals; however, this method is not yet suitable for routine use (Gharieb et al., p. 166 in [29]).

A series of studies has shown that livestock such as cattle, sheep, and goats remain the main zoonotic source of *C. parvum* infections in humans (Santín et al., p. 165 in [29]; Gharieb et al., p. 166 in [29]; Ongerth et al., p. 167 in [29]; Razakandrainibe et al., p. 168; p. 175 in [29]; Mammeri et al., p. 170 in [29]; Sahraoui et al., p. 171 in [29]; Bartley et al., p. 172 in [29]; Tesama et al., p. 178 in [29]). Subtyping of the 60 kDa glycoprotein (*gp60*) locus revealed that the majority of *C. parvum* infections in these livestock worldwide were caused by the IIa and IId subtypes (Mammeri et al., p. 170 in [29]; Sahraoui et al., p. 171 in [29]; Mravcová et al., p. 180 in [29]; Razakandrainibe et al., p. 175 in [29]). Following these studies, reports from the UK and Australia showed that adult cattle had more *C. parvum* genotype diversity than calves, and that *C. parvum* subtypes remain stable for long periods on the same farm (Shaw et al., p. 162 in [29]; Ongerth et al., p. 167 in [29]). The results presented at IGCC-7 also showed that adult cows do not represent the main source of *Cryptosporidium* infections for calves, as they are mostly infected from other calves (Shaw et al., p. 162 in [29]).

Although *C. parvum* subtype IIa, which is most prevalent in livestock, has been detected as a common pathogen in horses (Uiterwijk et al., p. 163 in [29]), wild ponies (Couso-Pérez et al., p. 71 in [29]), deer (Teixeira et al., p. 72 in [29]), and non-human primates (Wortea Hailu et al., p. 182 in [29]), all reports indicate that farm and wild mammals and birds are mostly parasitized by host-specific *Cryptosporidium* spp. In addition to the already known *Cryptosporidium* spp. in domestic and wild vertebrates – *C. suis* in pigs (Cervone et al., p. 174 in [29]), *Cryptosporidium* rat genotypes I and IV in rats (Kváč et al., p. 67 in [29]), *C. muris* and *C. tyzzeri* in mice (Kváč et al., p. 68 in [29]), *C. xiaoi* in sheep (Krumkamp et al., p. 111 in [29]), *C. bovis* and *C. ryanae* in cattle (Krumkamp et al., p. 111 in [29]), and *Cryptosporidium molnari*-like in fish (Gantois et al., p. 74 in [29]), novel genotypes have recently been reported: (i) a great tit genotype in great tit (Holubová et al., p. 67 in [29]); (ii) a swallow genotype in swallow (Holubová et al., p. 68 in [29]); and (iii) potentially novel genotypes (confirmation still pending) in trout (Couso-Pérez et al., p. 71 in [29]). These results are confirmation of the major trend of the last 10 years: there is still much to discover about the genus *Cryptosporidium*.

With the widespread use of molecular tools, *C. parvum*, the species most often associated with infections from water, has been increasingly detected in aquatic vertebrates. Studies from Poland, France, and Spain showed that turtles and fish can be passive carriers of *C. parvum* (Kaupke et al., p. 66 in [29]; Couso-Pérez et al., p. 71 in [29]; Gantois et al., p. 74 in [29]). Synanthropic rodents have also been reported as potential sources of *C. parvum* and *C. hominis* infections in human populations (Kváč et al., p. 69 in [29]). Conversely, a number of studies have shown *Cryptosporidium* transmission in the opposite direction. A study from Spanish zoos indicated the possible transmission of *C. hominis* and *C. parvum* from human (keeper) to non-human primates (Köster et al., p. 71 in [29]). Interestingly, various reports have described an increased frequency of detected cases of anthropogenic *C. hominis* in cattle (Krumkamp et al., p. 111 in [29]; Zahedi et al., p. 126 in [29]; Razakandrainibe et al., p. 175 in [29]) and kangaroos (Zahedi et al., p. 126 in [29]), as well as *C. viatorum* in cattle (Krumkamp et al., p. 111 in [29]). However, the role of animals in the transmission of *C. hominis* remains unclear, and the host range of *C. viatorum* is ill defined (Braima et al., p. 220 in [29]).

A study from the Czech Republic has shown that chickens infected as embryos with *C. baileyi*, a species that naturally infects chickens, or *C. parvum*, a species that is typically not infectious to chickens, could serve as a laboratory model for the propagation of *Cryptosporidium* isolates (Holubová et al., p. 180 in [29]).

## Human cryptosporidiosis and epidemiology

In some countries, cryptosporidiosis diagnosis and surveillance is well established, but it was clear from presentations at IGCC-7 that even in most high-income countries, there is still a need to promote prompt diagnosis (Loury et al., p. 114 in [29]; Mravcová et al., p. 180 in [29]; Bahadur et al., p. 125 in [29]; TuA-P9 Malika; Costa et al., pp. 153, 210, 213 in [29]). Varying molecular epidemiology at national levels was revealed at IGCC-7 from species identifications (Chalmers et al., p. 104 in [29]; Kortbeek et al., p. 115 in [29]). Sequencing the *gp60* gene was applied for surveillance and reported to be useful in outbreak investigations (Chalmers et al., p. 104 in [29]; Loury et al., p. 114 in [29]; Costa et al., pp. 110, 210 in [29]; Braima et al., p. 205 in [29]). This approach identified the introduction of novel subtypes by travellers. The development of bioinformatics tools for automatic genotyping of *Cryptosporidium* sequences was described (Yanta et al., p. 127 in [29]). Developments in multilocus genotyping (MLG) to improve discrimination over *gp60* sequencing were described (Jellison et al., p. 122 in [29]) and validation of an MLG scheme was provided (Pérez-Cordón et al., p. 119 in [29]). Evidence for MSC6-7 as a marker for bovine-derived *C. parvum* was presented (Razakandrainibe et al., p. 168 in [29]). Several human epidemiological studies used whole-genome sequencing, and its value in discovering the genetic diversity of *C. hominis* in low-income settings was demonstrated (Ajjampur et al., p. 212 in [29]; Tichkule et al., p. 120 in [29]).

The lack of specific treatment options for human cryptosporidiosis means that prevention of infection is paramount. Understanding transmission and identifying key intervention points can be improved by genotyping to identify species and subtypes. Descriptive studies reported at IGCC-7 identified links between unusual human infections, and sometimes exposure to known or possible host sources, *e.g.* chipmunk genotype 1, *gp60* XIVaA20G2T1, and squirrels (Beser et al., p. 206 in [29]), horse genotype and horses (Kopacz et al., p. 184 in [29]), and *C. erinacei* and hedgehogs (Costa et al., p. 210 in [29]). Wildlife sources of human infection need further investigation, but may present challenges to control. Household and neighborhood studies identified that, in some settings, zoonotic transmission is not as important as anthroponotic transmission, and that interventions for control should focus on sanitation and hygiene (Krumkamp et al., p. 111 in [29]; Eibach et al., p. 113 in [29]; Ajjampur et al., p. 212 in [29]). Such measures are important in schools where the potential for asymptomatic carriage was shown to increase the risk of infection in children (Köster et al., p. 208 in [29]; Muadica et al., p. 207 in [29]).

Clinical case reports are valuable, for example in identifying emergent, vulnerable patient groups, such as those being treated with biologics for rheumatoid arthritis (Kopacz et al., p. 184 in [29]). At IGCC-7, studies of long-term post-infection sequelae were presented, with cases reporting intestinal and extra-intestinal symptoms (Kortbeek et al., p. 117 in [29]; Carter et al., p. 116 in [29]). In a 12-month study, post-infection irritable bowel syndrome (IBS) or IBS-like symptoms were common, but there was no significant difference between *C. parvum* and *C. hominis* infection (Carter et al., p. 116 in [29]). It would be helpful in terms of patient management to advise patients of the likelihood of continued gastrointestinal symptoms for a prolonged period after a diagnosis of cryptosporidiosis. Further concern was raised from growing evidence of causal links between cryptosporidiosis and colon cancer, further highlighting the need for prevention and control (Certad et al., p. 101 in [29]).

## Genomes and genomic evolution

### Comparative genomics of *Cryptosporidium* spp.

Comparative genomics has been an active area in *Cryptosporidium* research in recent years [19]. Several IGCC-7 presentations reported recent data on the genomics of several common *Cryptosporidium* spp. Recent developments in the comparative genomics of *Cryptosporidium* spp., including the initial sequencing of the genomes of *C. parvum* and *C. hominis* 15 years ago, have revealed high genetic similarity between the two species with a very different host range (Cacciò, p. 97 in [29], [1, 11, 25, 43]). Recent advances discussed the development of procedures for direct isolation of oocysts from clinical specimens for whole-genome sequencing [2, 14, 16, 36], and the wide use of NGS techniques for high-throughput acquisition of sequence data [12, 13, 15, 18, 22, 24, 44]. These methods have led to improved understanding of intra-species genetic variations in the major human-pathogenic species *C. parvum* and *C. hominis* [15, 16, 22, 24], the identification of species-specific genes [15, 24], elucidation of the role of genetic recombination in the generation of host-adapted *C. parvum* and virulent *C. hominis* variants [15, 17], and development of better molecular epidemiological tools [7, 9].

Two presentations focused on genome evolution within *C. parvum* and *C. hominis*. Identification of the genetic determinants of host adaptation among different *gp60* subtype families of *C. parvum* using comparative genomics was discussed (Wang et al., p. 93 in [29]). Previously, the group reported whole-genome sequence differences between the bovine-adapted IIa subtype family and the small ruminant-adapted IId subtype family [12]. The recent whole-genome sequence analysis of over 300 *C. parvum* isolates from various sources expanded the observation of significant differences in nucleotide sequences among *C. parvum* subtype families. The genetic differences between the IIa and IId subtype families appeared to be much smaller than those between the IIa and anthroponotic IIc subtype families. Most highly polymorphic genes among the three subtype families were subtelomeric genes encoding secretory proteins, especially the invasion-associated and immunodominant mucin proteins and members of the *Cryptosporidium*-specific *SKSR* gene family. These subtype families also differed in the copy numbers of subtelomeric genes encoding the MEDLE family of secretory proteins and insulinase-like proteases. These observations are in agreement with the genomic differences reported between IIa and IIc subtype families by two other research groups [24, 41].

The genomics of the dominant *C. hominis* subtype IbA10G2 in European countries was examined (Alako et al. p. 96 in [29]). This subtype accounts for ~80% of human cases of *C. hominis* infections in this area, and is responsible for almost all outbreaks associated with *C. hominis* in Europe [8]. To understand the reasons for the prevalence of this subtype in Europe, these researchers sequenced the genomes of 19 IbA10G2 isolates from sporadic cases of cryptosporidiosis in the United Kingdom. This led to the generation of 7.4–13.8 million 150-bp, paired-end sequence reads per isolate, representing on average 112–214-fold coverage of the *Cryptosporidium* genome. Variant analysis of the whole-genome sequence data has identified sequence conservation among isolates, with higher sequence polymorphism in the subtelomeric regions of chromosomes 1, 2, and 6. This observation is similar to the findings reported recently by the Swedish group [34]. The authors concluded that there was clonal expansion of IbA10G2 in Europe. This is different from the population genetics of IbA10G2 in the United States, where genetic recombination is known to occur within the subtype due to the presence of other *C. hominis* subtypes within the country [15]. In Bangladesh, the high endemicity and subtype diversity of *C. hominis* has led to the extensive occurrence of genetic recombination within the species [13].

### Genomic insights into biology

Three talks highlighted biological insights gleaned from genomic analysis. The analysis of 21 whole-genome sequences revealed that *C. parvum* splits into two subclades, one of which is anthroponotic and consists of subtypes IIc-a (Nader et al., p. 123 in [29]). This lineage also shares a subset of loci with *C. hominis* that are also undergoing convergent evolution driven by positive selection. The analysis revealed genetic exchange between *Cryptosporidium* subtypes and identification of recombinant regions that are enriched for positively selected genes and potential virulence factors, indicative of recent, adaptive introgression, likely within the last millennium. Finally, an analysis of 467 *gp60* sequences revealed that the population structure of zoonotic and anthroponotic subtypes differed.

The genetic basis for virulence differences of various carcinogenic *C. parvum* isolates was examined (Audebert et al., p. 95 in [29]). The group has previously shown that the natural isolates of *C. parvum* named DID, TUM1 and CHR and all IIaA15G2R1 isolates are able to induce digestive adenocarcinoma in a rodent model, whereas *C. parvum* IOWA does not [6]. Whole-genome sequences of the three highly virulent carcinogenic *C. parvum* isolates were compared to the *C. parvum* IOWA II reference sequence. They found 126 common single-nucleotide variants in 91 coding sequences including several membranes and secreted proteins, as well as in previously characterized *Cryptosporidium* virulence factors implicated in sporozoite host cell invasion and intracellular multiplication/survival of the parasite. Their study has identified potential new virulence factors which will ideally be characterized using CRISPR/Cas9 technology [32].

Evidence has been found for developmental regulation of long noncoding RNAs (lncRNAs) and several other small ncRNAs in *Cryptosporidium parvum* (Li et al., p. 98, in [29]). This study utilized strand-specific RNA-Seq libraries of polyA- and non-polyA-selected fractions from different developmental stages. The authors discovered that ~20% of protein-coding genes are covered by transcription from the complementary strand, some of which are read through from neighboring genes. High-quality lncRNA candidates (>200 nt) revealed a subset with expression peaks 48 h post-infection. Some lncRNA candidates were conserved between *Cryptosporidium* species and even several Apicomplexa. Currently, their function is unknown. Further characterization of the *Cryptosporidium* non-coding transcriptome may provide further insights into parasite development and host-parasite interactions [23, 38, 39].

### Acquiring and utilizing genome sequences

New technologies are also making *Cryptosporidium* genome sequences accessible and usable. The development of an Agilent SureSelect target enrichment approach [10] for whole-genome sequencing of *Cryptosporidium* directly from DNA isolated from stool samples, with the goal of developing high-resolution genetic markers for genotyping, and characterizing asymptomatic carriage and the potential for zoonotic transmission was presented (Khan et al., p. 99 in [29]). The approach utilizes 75,000 probes that cover 95% of the genome to perform target enrichment. The captured DNA is sequenced with Illumina. The authors showed that 30% of the genome sequence can be recovered when as little as 0.01 ng of *Cryptosporidium* genomic DNA is present in the sample. When 5 ng is present, 98% can be recovered. The probes, which were designed using *C. parvum*, can capture degenerate sequences; they are therefore able to detect species as distant as *C. meleagridis*, although with somewhat lower efficiency.

The availability of consistent, comparative and evidence-based genome assembly and annotation for the three closely-related species *C. parvum*, *C. hominis*, and *C. tyzzeri* was presented (Baptista et al., p. 151 in [29], [31])*.* Finally, new data and tools available in the free online resources CryptoDB.org have been reported on behalf of EuPathDB [3] by Warrenfeltz et al. p. 27 in [29]. Since IGCC-6, new data include the Baptista genome assemblies and annotation and several new RNA-Seq and SNP data sets. Sequence data from 14 species and strains are now available [40].

### Genetic manipulation

*Cryptosporidium* can now be readily manipulated through CRISPR-driven recombination [37] and recent advances have simplified the required procedure. Remarkably, 50 base pairs are sufficient to serve as homology arms to drive site-specific modification [27], allowing the use of simple PCR products as targeting vectors. Furthermore, a simplified infection protocol that delivers sporozoites by gavage (after buffering of the stomach) has replaced surgery-based delivery [33]. These modifications have streamlined reverse genetics for *Cryptosporidium* and the method has been adopted by numerous laboratories in the meantime. At this conference, research based on genetic manipulation of the parasite’s sexual cycle, on the identification of a host-secreted family of proteins, and on the development of a mouse model of cryptosporidiosis to investigate host immunity was presented.

Genetic manipulation of *C. parvum* provided novel insights into the parasite’s sexual cycle. Using a combination of fluorescent reporters and epitope-tagged transgenic parasite lines, it was shown that parasites rapidly undergo sexual differentiation in culture, leading to the formation of male and female gametes (Striepen, p. 54 in [29], [35]). Male gametes were clearly identified by the expression of a *Cryptosporidium* hapless2 homolog (HAP2, cgd8_2220), and cell oocyst wall protein 1 (COWP1, cgd6_2090) proved to be a reliable marker of female gametes. Using a *C. parvum* transgenic strain that expresses the fluorescent protein tdTomato driven by the COWP1 female specific promoter, female gametes were isolated by fluorescence-activated cell sorting from both cell culture and from actively infected ${\mathrm{IFN}}_{\gamma }^{-/-}$ mice. Comparative analysis of gene expression by RNA sequencing showed that female gametes from cell culture and infected mice have very similar expression profiles, with upregulation of genes required for meiosis, wall formation, and oocyst persistence. However, female gametes from infected mice demonstrated an RNA expression profile that more closely aligned with the expression profile of sporozoites, suggesting that fertilization (and hence the production of new sporozoites) occurred *in vivo* but not *in vitro*. Follow-up experiments using a CRE recombinase-based assay to detect gamete fusion supported this hypothesis. Within mice, fusion, and fertilization were readily detectable by microscopy. However, in cell culture, no fusion or fertilization could be detected. This lack of fusion results in a lack of new oocyst production and, ultimately, a block of continuous parasite growth in cell culture (Striepen, p. 54 in [29]).

Secretory proteins delivered to the infected cell play important roles as effector molecules and are used by many pathogens to manipulate their hosts. Rapid coevolution of host and parasite drives high levels of polymorphism in the genes that encode these effectors. Using a bioinformatics approach, candidate host-secreted proteins were identified in *C. parvum* and subsequently tested by epitope tagging at their genetic loci using CRISPR-driven recombination. From this cohort of candidates, one protein localized to the cytoplasm of infected cells in both cell culture and in the intestinal epithelial cells of infected ${\mathrm{IFN}}_{\gamma }^{-/-}$ mice. This protein was not visible in sporozoites by immunofluorescent microscopy but could be easily detected in infected host cells, suggesting a delivery system following parasite invasion. Treatment of infected cells with Brefeldin A ablated export, suggesting that this protein passes through the canonical secretory pathway of the parasite to reach the host cell, traveling from the ER to the Golgi. The emerging model for effector export in *Cryptosporidium* identifies a specific amino acid sequence that distinguishes proteins destined for the host cell (Striepen, p. 54 in [29]).

Finally, the tools of genetic manipulation were also shown to extend to *C. tyzzeri*, a species that produces natural infection in wild and laboratory mice (Striepen, p. 54 in [29], [31]). A new isolate of *C. tyzzeri* was isolated in the United States (Athens, Georgia), and the genome was sequenced, assembled *de novo*, and fully annotated. Complete genomic alignment confirmed that *C. tyzzeri* is indeed closely related to the human infectious species, *C. parvum* and *C. hominis*, with 95–96% identity at the nucleotide level. Using a draft of the parasite genome, and CRISPR-driven homologous repair, a stable transgenic *C. tyzzeri* strain was created expressing both fluorescent and luminescent reporters. With this transgenic parasite line, it was shown that healthy adult mice develop a robust infection that is self-limiting, with intestinal pathology that mirrors human cryptosporidiosis. Following initial exposure to *C. tyzzeri*, mice then developed a marked resistance to subsequent infections. Further, this resistance could be replicated through immunization with gamma-irradiation attenuated parasites. Control of infection and the resistance to subsequent infections was shown to be both IFN*γ* and T-cell-dependent, again mirroring what is seen in human cryptosporidiosis. This natural murine model of cryptosporidiosis is anticipated to develop into an inexpensive platform for both vaccine development and drug testing (Striepen, p. 54 in [29]).

## Host-parasite interaction (immunology, microbiome)

Investigating host-parasite interactions to better understand parasitic infection and survival within the host is a prerequisite to develop strategies for prevention and treatment of infectious diseases. Recent advances in the genetic manipulation of *Cryptosporidium* combined with new *in vitro* and *in vivo* models will allow a better understanding of these interactions.

It is now well known that *Toxoplasma gondii* organelles such as rhoptries or dense granules store many effector proteins targeting host signaling and transcription [17]. It was also clearly demonstrated that *C. parvum* also exports proteins into the cytoplasm of the epithelial host cell (Dumaine et al., p. 100 in [29]. *MEDLE2*, a gene that belongs to a polymorphic gene family, is produced during the course of the infection soon after the parasite makes contact with the intestinal epithelial cell and is exported to the cytoplasm. This process occurs at all stages of the parasite (asexual and sexual stages) and requires the presence of a signal peptide and specific sequence information that directs the export of the MEDLE2 protein into the host cell. Although the precise function of this protein is not yet known, genes in the unfolded protein response pathway are upregulated in cells that express MEDLE2. Dumaine’s elegant study paves the way for the characterization of *C. parvum* virulence factors that modulate host metabolism to the parasite’s benefit, or help the pathogen escape from the immune response (Dumaine et al., p. 100 in [29]).

The range of tools to investigate the immune response to *C. parvum* infection and the host-pathogen interaction has expanded. For some investigations, mouse models can present limitations. For example, in contrast to neonatal mice, adult mice are resistant to *C. parvum* infection and there was thus no suitable immunocompetent adult mouse model to study *Cryptosporidium* infection. To fill this gap, Sateriale et al. isolated a line of *Cryptosporidium tyzzeri*, a species that naturally colonizes the small intestine of mice and is also genetically tractable with CRISPR/Cas9 (Sateriale et al., p. 46 in [29], [31]). With this new model and several transgenic mouse lines, it was possible to confirm in adult mice that interferon *γ* acts as a key cytokine and that T cells are important for the resolution of the infection. More importantly, this study demonstrated that primary infection protects against a homologous challenge, reviving interest in developing a vaccine to prevent or limit the severity of cryptosporidiosis. Major improvements in the ability to follow the progression of the *Cryptosporidium* life cycle were presented. This was accomplished by generating several transgenic lines expressing a reporter controlled by stage-specific promoters, such as promoters active during sexual differentiation and oocyst formation. This work also highlighted the limitation of the classical *in vitro* culture methods using epithelial cell lines. Evidence was presented that male gametes are able to find macrogamonts but do not fertilize them efficiently. In this context, although not presented at the meeting, readers may be interested in the recent publication by Wilke et al. [42] who describe an air-liquid-interface cultivation system that supports life-cycle completion and *de novo* production of oocysts that can infect mice. This research constitutes a breakthrough to investigate genetic crosses *in vitro*. It is also a valuable tool for studying host-parasite interactions and to maintain stable *Cryptosporidium* strains. The precise mechanism by which *C. parvum* adheres to the surface of epithelial cells and successfully invades the host cell is still unknown. Three TSP1 domains containing proteins (TSP3, TSP4, and TSP7) localized on the surface of sporozoites and merozoites that bind to HCT8 cells in a dose-dependent manner were identified (Yin et al., p 35 in [29]). Further investigations suggest that these proteins could bind to heparan sulfate on host cells.

Our knowledge of the immune response to cryptosporidiosis is constantly improving. This research is of major importance to develop targeted immunostimulation strategies and vaccines. An overview on how innate responses shape *Cryptosporidium* infection with a particular focus on the role of mononuclear phagocytes was presented (Laurent et al., p. 38 in [29]) [20]. Previous findings highlighted the key role of dendritic cells in the control of the acute phase of the infection. Further investigations with transgenic mice revealed that a specific subset of dendritic cells, called cDC1, that express the transcription factor Batf3, plays a major role in the control of the acute phase of the infection, but also in the definitive clearance of the parasite [28]. These cells have the ability to produce large amounts of IL-12 during the course of infection but also interferon *γ* that might activate larger local producers of interferon *γ*, such as ILC1 and TH1 cells, for efficient control of *Cryptosporidium* development in epithelial cells. However, not all mononuclear phagocytes are involved in protection and it was shown that inflammatory monocytes (Ly6c+) play no role in protection, but participate in the loss of intestinal permeability by producing large amounts of IL1β in close contact with infected epithelial cells. This process was shown to destabilize cellular junctions. Paneth cells (PCs), specialized epithelial cells that produce antimicrobial peptides at the base of the intestinal crypt, are known to be active against free *C. parvum* stages (Lacroix-Lamandé et al., p. 33 in [29]). Neonatal mice deficient for PCs presented higher levels of infection, and the number of granule-positive PCs and lysozyme-positive PCs in infected conventional mice decreased, thus supporting the view that antimicrobial peptides including lysozymes participate in the efficiency of the innate immune response. As an escape mechanism, *C. parvum* can limit some of these effectors, such as CRAMP, an antimicrobial peptide specifically expressed in the neonatal intestine.

Enteric infections in early life can alter the development of the intestinal immune response and the establishment of the intestinal microbiota. This process can have long-term consequences on gut health. Epidemiological studies have reported that after resolution of *C. parvum* infection, patients still suffer from abdominal pain. Long after recovery, mice presented an alteration of immune cell composition and microbiota associated with increased visceral susceptibility (Lacroix-Lamandé et al., p. 34 in [29]). In addition, mice infected with *C. parvum* at a young age were more susceptible to *Salmonella* infection as adults, thus highlighting the detrimental role of early cryptosporidiosis on long-term gut health and susceptibility to enteric diseases. In addition, there is accumulating evidence that *C. parvum* infection could be associated with the development of digestive neoplasia. In a corticoid dexamethasone-treated adult severe combined immunodeficient (SCID) mouse model, cryptosporidiosis is associated with the presence of digestive adenocarcinoma (Certad et al., p. 101 in [29]). Neoplastic lesions were repeated with a 3D culture model from the adult murine colon maintained *ex vivo* for a month. This original model allowed the researchers to emphasize the pivotal role of the Wnt pathway together with the alteration of the cytoskeleton [5].

Research on host-parasite interaction has typically been the realm of immunologists. Particularly with respect to enteric pathogens, this view is changing, as is the definition of host. The improved understanding of the many functions of the abundant microbial population living in the gastro-intestinal tract, as well as other organs, has expanded the definition of host to a multi-species consortium. In terms of number of cells and metabolic function diversity, this multi-species consortium has been shown to be dominated by commensal bacteria. Consistent with this changing perception of the host, a field of research focused on understanding the interaction of the intestinal microbiome with enteric protozoa, like *Cryptosporidium*, *Giardia*, and *Entamoeba* is emerging. *Cryptosporidium parvum* infection in neonatal mice induced lasting dysbiosis (Lacroix-Lamandé et al., p. 33 in [29]). Research on the interaction between the intestinal microbiome and *Cryptosporidium* conducted in Giovanni Widmer’s laboratory at Tufts University was the focus of two talks. The interaction between the intestinal microbiome in mice and the proliferation of *C. parvum* and *C. tyzzeri* was investigated (Widmer et al., p. 92 in [29]; Oliveira et al., p. 45 in [29]). By enumerating the output of oocysts in the feces, the authors explored the effect of nutritional interventions on the severity of cryptosporidiosis. Probiotics, prebiotics, and dietary fiber can affect the severity of the infection, albeit in different ways. Mice fed prebiotics or dietary fiber developed a less severe infection than control mice fed no prebiotics or diet lacking fiber, respectively. In contrast, probiotics containing live *Lactobacillus*, *Bifidobacterium*, and *Streptococcus* bacteria were shown to have a opposite effect. Mice receiving this dietary supplement excreted significantly more oocysts over the course of the infection. 16S amplicon sequencing was used to investigate the effect of the dietary treatment on the intestinal microbiome and to identify bacterial taxa which correlate in abundance with parasite proliferation. Given the lack of effective drugs to control cryptosporidiosis, nutritional interventions could play a beneficial role in mitigating the effect of cryptosporidiosis, particularly in infants living in low-income countries where cryptosporidiosis is highly prevalent [25, 26].

## Diagnosis and treatment

During human diarrhea outbreaks, countries such as France prioritize the search for bacterial or viral agents, and a parasitological examination of stools is only carried out with a long delay, making the availability of samples very random. In order to have a simple test allowing *a posteriori* diagnosis of cryptosporidiosis, a simple test based on the search for antibodies in the saliva of patients was developed and then evaluated (Elwin et al., p. 196 in [29]). They showed that the results obtained with these saliva samples are correlated with those obtained by testing for antibodies in the serum, and that antibody levels peak 60 days after contamination and then disappear in the months that follow. Regarding the coprological diagnosis, a large number of recently marketed multiplex tests have been evaluated, and their performance found to be satisfactory with regard to the detection of *Cryptosporidium* (Basmaciyan et al., p. 231 in [29]; Lamien Meda et al., p. 195 in [29]; Costa et al., p. 200 in [29]; Razakandrainibe et al., p. 201 in [29], [4]).

Cryptosporidiosis is a leading cause of diarrheal disease. There remains a critical need for new approaches to the treatment of this infection. There are no vaccines and nitazoxanide (NTZ) is the only drug approved for the treatment of cryptosporidiosis. Basic research to drive new molecule development has stagnated mainly because of the lack of genetic tools and suitable animal models. The pace of progress in these areas is expected to increase in the next decade, especially fueled by the completion of several *Cryptosporidium* genome sequences and the new-found ability to genetically engineer *Cryptosporidium*, making it possible to non-invasively track parasite tissue burden throughout the gastrointestinal tract (Gargala p. 234 in [29]). As there is no continuous culture for *Cryptosporidium*, investigators have relied on short-term maintenance in cultured cell monolayers to assess the efficacy of drugs. Oligoarginine synthetically attached to NTZ (NTZ-CPP) induced a drastic improvement of drug activity as compared to NTZ. These results are highly attractive and represent an encouraging step toward developing an alternative therapy to NTZ (Nguyen et al., p. 190 in [29]). The routes of drug uptake are still poorly known and it is unclear whether, for intestinal cryptosporidiosis, the optimal anticryptosporidial agent should be absorbed systemically and/or retained in the gastrointestinal tract. Ideally, the treatment of extra-digestive or severe cryptosporidiosis calls for an injectable compound. Aminoxanide, a new amino-ester thiazolide derivative and the first soluble form of NTZ was identified as a candidate (Diawara et al., p. 238 in [29]). Developing therapeutics to reduce morbidity and mortality due to *Cryptosporidium* spp*.* among children in low-resource settings is a challenge (De Hostos et al., p. 237, in [29]), and a study has been conducted aiming to explore the traditional Ivorian pharmacopoeia to identify new effective compounds against *C. parvum*. In this context, an ethnobotanical survey focussing on anti-diarrheal therapeutic habits led to the evaluation of the anticryptosporidial effect of plant extracts and found that 6 out of 28 extracts showed greater efficacy than paromomycin (Tuo et al., p. 192 in [29]).
